# Chimpanzees Show a Developmental Increase in Susceptibility to Contagious Yawning: A Test of the Effect of Ontogeny and Emotional Closeness on Yawn Contagion

**DOI:** 10.1371/journal.pone.0076266

**Published:** 2013-10-16

**Authors:** Elainie Alenkær Madsen, Tomas Persson, Susan Sayehli, Sara Lenninger, Göran Sonesson

**Affiliations:** 1 Department of Cognitive Science, Lund University, Lund, Sweden; 2 Department of Semiotics, Centre for Languages and Literature, Lund University, Lund, Sweden; 3 Department of Linguistics, Centre for Languages and Literature, Lund University, Lund, Sweden; 4 Humanities Lab, Lund University, Lund, Sweden; Max Planck Institute for Evolutionary Anthropology, Germany, Germany

## Abstract

Contagious yawning has been reported for humans, dogs and several non-human primate species, and associated with empathy in humans and other primates. Still, the function, development and underlying mechanisms of contagious yawning remain unclear. Humans and dogs show a developmental increase in susceptibility to yawn contagion, with children showing an increase around the age of four, when also empathy-related behaviours and accurate identification of others’ emotions begin to clearly evince. Explicit tests of yawn contagion in non-human apes have only involved adult individuals and examined the existence of conspecific yawn contagion. Here we report the first study of heterospecific contagious yawning in primates, and the ontogeny of susceptibility thereto in chimpanzees, *Pan troglodytes verus*. We examined whether emotional closeness, defined as attachment history with the yawning model, affected the strength of contagion, and compared the contagiousness of yawning to nose-wiping. Thirty-three orphaned chimpanzees observed an unfamiliar and familiar human (their surrogate human mother) yawn, gape and nose-wipe. Yawning, but not nose-wiping, was contagious for juvenile chimpanzees, while infants were immune to contagion. Like humans and dogs, chimpanzees are subject to a developmental trend in susceptibility to contagious yawning, and respond to heterospecific yawn stimuli. Emotional closeness with the model did not affect contagion. The familiarity-biased social modulatory effect on yawn contagion previously found among some adult primates, seem to only emerge later in development, or be limited to interactions with conspecifics. The influence of the ‘chameleon effect’, targeted vs. generalised empathy, perspective-taking and visual attention on contagious yawning is discussed.

## Introduction

Contagious yawning (henceforth, CY) is well-established in humans [1]–[5]. Viewing videos of others yawning elicits CY in approximately half of adults, and the thought [1] and sound [5] of yawning is sufficient to elicit contagion. Yawn contagion has also been reported in chimpanzees, *Pan troglodytes spp.* [6]–[8], bonobos, *Pan paniscus* [9], gelada baboons, *Theropithecus gelada* [10], domestic dogs, *Canis lupus familaris* [11], [12] and budgerigars *Melopsittacus undulatus* [14]. While CY has been reported for stumptailed macaques, *Macaca arctoides*, elevated levels of concomitant self-directed scratching, suggested that the yawns derived from tension, rather than contagion [15]. The only solitary species tested for CY, the tortoise, *Geochelone carbonaria* [16] has shown no evidence of contagion. While the ultimate function of yawning remains disputed (for a review, see [17]), it has been suggested to carry thermorgulatory [18] and non-verbal communicative functions, and its contagiousness to serve the adaptive function of synchronizing group behaviour [19], with respect to arousal [20] and attention [21]. On a proximate level, the facial expressions that individuals adopt, tend to influence their emotional experiences (e.g. [22], [23]), suggesting that yawn contagion allows individuals to automatically mimic and synchronise facial expressions and movements with others, and consequently converge behaviourally and emotionally [24].

Research to uncover the underlying mechanisms of CY has suggested that it may be linked to and modulated by empathy (e.g. [4], [7], [9], [10], [11], [13], [24], [25]), represent a case of non-conscious mimicry (the ‘chameleon effect’) [27] or a fixed action pattern [1]. Empathy refers to a spectrum of interacting emotional and cognitive reactions to the experiences of others, and is mediated by at least two separate systems: the ability to *feel* and to *imagine* others’ emotional experiences. The ability to feel others’ emotional experiences (‘*affective empathy*’) derives from a phylogenetically old social contagion system, whereby one is viscerally affected by another’s emotional or arousal state (e.g. [28]). *Affective empathy* is a largely automatic process and may come about through *emotional contagion*, whereby the perception of expressive behaviour transfers emotional states from one individual to another (i.e. the tendency to ‘catch’ emotions from observed emotional states of others: [29], see also [30], [31]). In contrast, the ‘*cognitive empathy* system’ (also termed empathetic perspective-taking: [28]) entails *imagining* another’s emotional experience. This system emerges phylogenetically and ontogenetically with other ‘indicators of mind’ and requires a capacity for self-other differentiation, perspective-taking and mental state attribution, without necessarily resulting in emotional matching [28], [32]–[37]. There is ample evidence that the affective and cognitive components of empathy dissociate in humans and have different developmental trajectories, and that *affective empathy* precedes *cognitive empathy*, ontogenetically and phylogenetically [33], [38]–[40].

In human adults CY correlates with self-reported measures of empathy and is positively related to visual self-recognition and performance on theory-of-mind tasks [4], abilities considered constituent parts of *cognitive empathy* (e.g. [36]). Furthermore, research has indicated that CY is reduced in individuals with empathy-related disorders (e.g. schizotypy [4] and autism [2], [41], [42]). Recent evidence suggests that *affective* and *cognitive empathy* may dissociate in psychopathological populations, such as individuals with autism spectrum disorder (ASD), who seem primarily impaired in *cognitive*, but not *affective*, empathy [38], [39]. Since autistic children have reportedly failed to evince CY [2], [41], [42], this has contributed to the view that CY is linked to *cognitive empathy* and theory-of-mind capacities (e.g. [4], [43]). Recent research has, however, shown that when children with ASD have been instructed to fixate on a yawner’s eyes, they yawn contagiously with a frequency equal to that of typically developing children ([44] see also [45]). The previously found association between yawn contagion, autism, and failure on tests of *cognitive empathy* and theory-of-mind may therefore be one of correlation, rather than causation, and rely on differences in attentional states. Moreover, while the association between mental state attribution and CY in adults [4] and the temporal emergence of CY in children (around 4 years [3], [42], [46]) is consistent with the suggestion that CY shares a basis with *cognitive empathy* and theory-of-mind [4], [43], evidence of CY in a number of non-human species [6]–[14], not typically associated with *cognitive empathy*, suggests that the phenomenon is underlain by lower-level processes.

Alternatively, CY may be unrelated to empathy *per se,* but rely on non-conscious mimicry, also termed the ‘chameleon effect’ [27]. The ‘chameleon effect’ refers to an individual’s tendency to mimic a social partner’s behaviours (postures, gestures and facial expressions etc.) without either individual’s awareness or intent [47]. The ‘chameleon effect’ has affective and behavioural consequences for the subsequent interactions of the involved individuals in terms of increased levels of affinity, liking, empathy and prosocial behaviours [48]–[50]. Moreover, conversely, in humans social motivations, such as the desire to affiliate or bond with another, modulate non-conscious mimicry [47]. Studies thus converge to suggest that mimicry serves a prosocial function (to smooth social interaction), and that the relationship between non-conscious mimicry and affiliation is bidirectional: non-conscious mimicry fosters affiliation, and affiliation fosters non-conscious mimicry [51]. There is evidence that the ‘chameleon effect’ is not limited to humans, as capuchin monkeys (*Cebus apella*) affiliate more with humans, who have previously imitated them [52]. Moreover, the ‘chameleon effect’ operates in children as young as 18 months of age, for whom being mimicked increases pro-social behaviour [50]. CY however, does not emerge in children until around 4 years of age, suggesting that the ‘chameleon effect’ is unlikely to underlie contagious yawning *per se*. The ‘chameleon effect’ may nonetheless modulate CY, once the capacity is in place.

An even lower level mechanism, a fixed action pattern [53] has been suggested to underlie CY [1]. According to this hypothesis, CY relies on a specific, fixed and unlearned behavioural action pattern, for which the releasing stimulus is another’s yawn. The hypothesis is supported by the fact that yawning follows a stereotyped pattern [1] and may be triggered by multiple (minimal) forms of stimuli independently (e.g. observing, hearing and thinking about others yawning [1]–[5]). While fixed action patterns may have moderators, evidence that CY is modulated by a social variable (familiarity/social bonding [7], [9], [10], [13], [26]), has led to the suggestion that CY requires more complex mechanisms. Nonetheless, the fixed action pattern, empathy and ‘chameleon effect’ hypotheses are not mutually exclusive explanations for the mechanisms underlying CY.

Adult humans, chimpanzees, bonobos and gelada baboons have shown a social modulatory effect on the strength of contagion. For humans cross-cultural observational data have shown the CY effect to be stronger in response to the yawns of kin, then friends, then acquaintances, and lastly strangers [26]. Similarly, for bonobos and gelada baboons, CY correlates with social bonding [9], [10]. Studies of chimpanzees present a more complex picture. Chimpanzees yawn contagiously in response to videos of yawns by familiar, but not unfamiliar conspecifics (i.e., non-group members [7]). In contrast to bonobos [9], baboons [10] and humans [26]), chimpanzee susceptibility to yawn contagion does, however, not appear affected by relationship quality with *familiar* conspecifics (as indexed by grooming and proximity patterns [8]). While this may suggest that yawn contagion in chimpanzees is influenced by familiarity (group membership), but not relationship quality (with in-group members), the methodologies used may account for the differences found across species. While the positive effect of relationship quality on CY in bonobos, baboons and humans has been established through observational studies of spontaneous yawns, the negative findings for chimpanzees derived from projecting videos of yawning group-members on a wall. Videos were presented to multiple individuals simultaneously, including sometimes to the individual depicted in the video. The results may thus have been influenced by the medium, the attentional states of observing chimpanzees, and the likelihood of others yawning to the stimuli. In contrast the study, in which chimpanzees were found to yawn contagiously to familiar, but not unfamiliar, conspecifics, presented video stimuli individually, in a context where attentional focus was ensured [7]. The order of presentation of the in- and out-group chimpanzee yawn stimuli may, however have produced carry-over effects, as all subjects first viewed videos of familiar in-group members yawning, meaning that less attention may have been paid to the out-group yawn stimuli, viewed at a later point. The difference in CY to familiar and unfamiliar conspecific yawns may thus lie in attentional states, and the results await additional analyses or replication. Ascertaining whether CY in chimpanzees is influenced by relationship quality, or only by a less fine-grained in-group/out-group effect requires further research.

The literature on a potential social modulatory effect on CY in dogs is also contradictory, with the authors of one study suggesting that dogs exhibit auditory CY to the sound of only *familiar* yawns [54] (for a criticism of the study, see [12]), while other studies have demonstrated CY to strangers during live interactions [11], [12], and found no evidence of familiarity-biased contagion in young [12] and adult dogs ([55], for a methodological criticism of this study, see [13]). A recent study, in which dogs were tested under low-stress conditions, however suggests that for adult dogs CY is correlated with level of emotional proximity to the model [13].

Overall, as predicted by the perception-action model [33], studies suggest that familiarity increases state-matching [33], [56] and for primates, contagious yawning [7], [9], [10], [26]. While, however, the familiarity effect on CY has broadly been interpreted as support for the hypothesis that CY is modulated by empathy, the studies reviewed cannot distinguish whether the modulating variable for CY is empathy (operationalised as degree of social bonding with the original yawner) or the ‘chameleon effect’ (non-conscious imitation to smooth social interaction and cement relationships).

Thus far, social modulation of yawn contagion has only been demonstrated in adult individuals. In neither human children [46], nor young domestic dogs [12] is the emergence, or strength of yawn contagion influenced by familiarity with the yawning model, which has lead to the suggestion that the social modulatory effect in adult primates (including humans) only emerges at later stages of development (‘developmental hypothesis of empathy modulation of CY’ [12]).

Humans and dogs show a developmental trend in susceptibility to yawn contagion, which does not become prominent until around 4 years of age in humans [3], [42], [46] and 7 months in dogs [12]. Naturalistic observations suggest a similar developmental trend in gelada baboons, for which a study found that four infants exhibited few yawn responses to nearby adults’ yawning [10]. In humans CY emerges developmentally at the time when also cognitive-empathy-related behaviours begin to clearly manifest, and children begin to show an increase in the ability to correctly identify the emotions of others (e.g. [57]–[61]). Thus far, only chimpanzees above the age of 10 yrs. have been explicitly tested for yawn contagion, with some suggestion of a potential age effect. This indication derives from a study, in which videoed stimuli of conspecific yawns elicited CY in only the two oldest individuals tested (26 and 27 yrs.), while not in four younger individuals (21–26 yrs.), nor in any of three infants accompanying their mothers to the test [6].

All previous tests of CY in chimpanzees have deployed videoed [6]–[8] or computer animated stimuli of conspecific yawns [62]. There is, however, some indication that the medium may mask the ‘message’ for younger and non-human subjects. For example, live models have elicited CY in four-year-old children (35% of 4 yr. olds tested [42]), while neither videos, nor stories, in which the protagonist repeatedly yawned, have evoked CY in children below 5 yr. [3]. Moreover, while dogs have shown CY in three of four experiments involving live models [11], [12], [13], [55], they have failed tests involving videoed (conspecific and human) yawn stimuli [55], [63].

Some authors have suggested that differences in susceptibility to yawn contagion may owe to an attention bias, whereby observers pay closer attention to affiliated familiar individuals than non-affiliated ones [27]. While attention differences have been proffered to account for the apparent association between yawn contagion and empathy (operationalized as social bonding, relationship quality and familiarity: [7], [9], [10], [13], [26]), it also applies to findings of a developmental progression of susceptibility to CY in children [3], [42], [46] and dogs [12]. Young individuals may simply pay less attention to others’ physical and emotional states, than older individuals do.

In this study we examined the extent to which two factors affect chimpanzees’ susceptibility to yawn contagion: ontogeny (their age, infants and juveniles), and emotional closeness to the yawning model. While attention levels are difficult to certify and quantify, we ensured that yawns were perceivable, by presenting them dependent on the chimpanzees’ attentional focus (and repeating yawns if initially presented outside the chimpanzees’ field of vision). We sought to increase ecological validity and the chance of evoking CY in younger subjects by using live rather than videoed models. For practical reasons we consequently used human models, and the study thus represents the first test of interspecies yawn contagion in primates. We hypothesised that, if CY is related to the development of empathy and emotional understanding in humans, a similar developmental effect might be found in chimpanzees. We therefore predicted that (1) juveniles would be more susceptible to CY than infants. As model identity and empathy may facilitate social behaviours, such as non-conscious facial mimicry [64] and imitation [65]), we presented the chimpanzees with a familiar yawning model, that they had a strong and positive emotional relationship with (their surrogate human mother) and an unfamiliar model. We predicted that (2) the chimpanzees would be more likely to yawn contagiously to the familiar than unfamiliar model, and that the familiar model would evoke more CY in the infants. We moreover predicted (3) yawn frequency to increase in response to viewing a human model performing repeated yawns, but neither of two control behaviours, nose-wiping and gaping, nor when the model performed none of the three behaviours (baseline phase). Previous studies have deployed various control behaviours (gapes, laughs, smiles and coughs, see [66]). Our key control behaviour, gaping, however has the advantage of including much of the motor pattern of a yawn, while remaining an arbitrary expression. If CY is an emotional contagion [24], reflecting perception and internalisation of the emotion and/or physical state that another’s yawning reflects (for anecdotal observations of this in dogs, see [12]), only yawn stimuli should evoke yawning. A comparison of the rate of yawing in response to yawn and gape stimuli thus goes some way to exclude a more motoric, reflexive interpretation of CY. We also examined the chimpanzees’ responses to viewing a human nose-wipe, as this is a facially oriented action, which occurs frequently and spontaneously in chimpanzee behaviour. Since it can be a marker of nervousness [67], or itchiness, it may also correlate with potentially contagious emotions. While there is some suggestion that behaviours other than yawning are also contagious (e.g. laughter in chimpanzees [68] and humans [69], itching in humans [70], [71] and stretching in budgerigars [14]), there is no empirical evidence to suggest that such behaviours are underpinned by individual levels of empathy, or empathy with the observed model [71]. It is, however, conceivable that such effects may be underpinned by lower-level processes, such as a perception-action mechanism [33] or proto-mimesis (the matching of exteroception and proprioception based on mirror-neuron systems: [72]). We therefore remained agnostic, as to whether a potential contagion effect for yawning and nose-wiping would be similar or different. Finally, we strove to minimise the possibility of evoking ‘tension yawns’ by engaging the chimpanzees in a bouts of calm play and cuddling through the bars of their enclosure.

## Methods

### Participants

Participants were 33 orphaned chimpanzees (*Pan troglodytes verus*), 12 infants (13 months –4 yr., mean age ± SD = 2.83±1.19 yr., 6 females, 6 males) and 21 juveniles (5–8 yr., mean age = 7.02±0.89 yr., 9 females, 12 males, see Supplementary Materials, Table S1). Data was recorded dichotomously (as deriving from infants and juveniles), due to lack of access to sufficient numbers of chimpanzees, to compare the developmental trajectory of yawn contagion at individual ages (see Suppl. Matr., Table S1). The chimpanzees were housed at Tacugama Chimpanzee Sanctuary in Sierra Leone, where they had spent between 4 weeks and 6 years (infants: mean = 1.47±0.90 yr., range = 2.75 yr.; juveniles: mean = 4.73±0.87 yr., range = 3.33 yr.) having arrived between the ages of 1 month and 4.50 years (mean = 1.95±1.11 yr.). Most participants had been separated from their mothers as part of the illegal pet and/or bushmeat trade. The sanctuary followed the rehabilitation strategy of PASA (Pan African Sanctuary Alliance), and the chimpanzees were given a rich physical and social environment to promote the expression of species-typical behaviours, and potential release back into the wild. Infants were initially placed in quarantine and later introduced into peer nursery groups. Throughout their time at the sanctuary they were provided with a surrogate human mother, who comforted, carried, helped feed, and occasionally rested with the chimpanzees in their cages. Seventy-nine per cent (n = 26) of participants were housed in groups of 14 and 22 conspecifics in large outside enclosures (of 332 m^2^ and 969 m^2^) that contained natural tropical forest foliage, edible plants and trees for climbing. The chimpanzees were housed in a number of smaller outdoor cages (25 m^2^) during the night. The remaining 21% (n = 7) were infants that had arrived at the sanctuary within 12 months and were housed either alone (n = 5) or in a dyad (n = 2). All the chimpanzees were experimentally naïve, and only animals, that did not exhibit overt signs of stress from the separation from conspecifics, participated in trials. One participant was excluded due to fussiness and one due to refusal to engage with the models.

Studies suggest that laboratory chimpanzees separated from their mothers before the age of two and housed without access to peers, or in fairly sterile physical environments, may exhibit long-term aberrant behaviours [73]. A comparison of the cognitive abilities of orphaned sanctuary infants and mother-reared chimpanzee infants on a range of cognitive tasks has, however, shown that the socio-cognitive abilities of sanctuary orphans are comparable to those of mother-reared, same-aged infants living at a zoo [74]. Moreover, sanctuary orphans exhibited lower rates of aberrant behaviours (coprophagy, faecal smearing and rocking) than chimpanzees at a modern zoo facility with an enrichment program [72].

### Design and Procedure

The study deployed a repeated measures design, with one classification variable (age category: infant or juvenile) and two independent variables: model familiarity (unfamiliar and familiar human model: a female researcher and the chimpanzees’ surrogate human mother since their arrival at the sanctuary), and model behaviour (none/baseline, yawning, gaping, nose-wiping). Each participant received seven 5-min phases presented in immediate succession (for a total duration of 35 min). A trial sequence consisted of a baseline phase followed by three experimental phases, where the model repeatedly either yawned, gaped or nose-wiped. Each phase was followed by a five-minute post-stimulus observation interval, during which social interaction continued, without the inclusion of the key behaviours (yawning, gaping, nose-wiping). The chimpanzees were tested, individually, between 8.30 h and 16.30 h in an outdoor cage, that otherwise served as their sleeping quarters. In all phases the model encouraged the participant to engage in calm interactions (hugging, grooming and playing) through the bars of the enclosure.

Yawning phase: The model repeatedly yawned while being within the participant’s ‘full’ or ‘peripheral’ field of vision (defined, respectively, as the visual field measured 0–45° and 45–110° from the saggital plane between the animal’s eyes). Models yawned as naturally as possible, with yawning defined as opening the mouth fully, drawing in air, lifting the shoulders, tilting the head and body backwards, closing the eyes, and producing a vocalisation during exhalation, for a total duration of 5–10 s. (see Suppl. Matr., Video S1 for demonstrations of the conditions).

Gaping phase: The model performed repeated (non-yawning) mouth openings, i.e., opening the mouth widely and closing it, without audible inhalation and exhalation of air (approx. 4 s duration), while within the participant’s ‘full’ field of vision.

Nose-wiping phase: The model repeatedly wiped her hand over her nose (approx. 3 s duration), while within the participants’ ‘full’ field of vision.

Models aimed to expose participants to 15 instances of each behaviour, yet given the restive nature of the participants, models often produced the three behaviours outside the participants’ field of vision. In such cases, the behaviours were repeated, while within the participants’ ‘full’/‘peripheral’ field of vision, and the number of yawns, gapes and nose-wipes performed by models therefore varied across participants (X ± SE: yawning: 16.76±0.47, range = 18, gaping: 28.31±1.13, range = 39, nose-wiping: 30.91±1.24, range = 43). The number of yawns (Independent t test: *t_(_*_33,1) = _0.13, *P* = 0.90), nose-wipes (*t*_(32,1)_ = 1.34, *P* = 0.18), and gapes (*t*_(25,1)_ = 0.57, *P* = 0.57) made by the two models did, however, not differ significantly, nor was there a relationship between the number of presented and elicited yawns (familiar model: Spearman’s test: *r = *0.18, *P* = 0.32, unfamiliar model: *r* = 1.63, *P* = 0.36) and nose-wipes (familiar model: *r* = 0.30, *P* = 0.10, unfamiliar model: *r* = 0.34, *P* = 0.10). (These analyses of nose-wipes and gapes were based on, respectively, 32 and 25 trials – rather than 33 trials – as the number of model behaviours could not be reliably coded in, respectively, one and eight trials (as the model at times faced away from the camcorders). All conditions (except for the baseline phase) were counterbalanced across participants, including trials with the familiar and unfamiliar models (see Suppl. Matr., Table S2 for details). Participants received two full trial sequences, one with the familiar and unfamiliar model, with a minimum 24-hour interlude. Yawning and nose-wiping occurrences were scored from videos of the trials (recorded by two Panasonic camcorders, HDC-HS700S and HDC-SD700).

#### Analysis

Both individual phases (yawn, nose-wipe, gape) and the subsequent five-minute post-observation phases were used as means of comparisons (i.e., the yawning and post-yawning, as well as nose-wiping and post-nose wiping, and gaping and post-gaping phases were collapsed). Trials from 21% of a random selection of participants were scored for interrater reliability, which was perfect with respect to number of chimpanzee yawns (100% agreement) and high with respect to nose-wipes (Cohen’s kappa = 0.94). We used independent t-tests to assess differences in the number of yawns, nose-wipes and gapes produced by models, and Spearman’s correlations to assess the relationship between numbers of presented and elicited yawns and nose-wipes. Data were analysed at both individual level (number of chimpanzees that yawned and nose-wiped across conditions, binomial analyses) and group level. For the latter, the number of yawns and nose-wipes per minute were used, as the duration of the compared baseline, yawn, nose-wipe and gape conditions differed. Generalised linear mixed models (GLMM) were used to assess the effect of model behaviour, model familiarity and participant age on yawns and nose-wipes, and interaction effects. We controlled for participant identity (random effect) and used robust covariances and a Satterhwaite approximation (due to the small sample size). Friedman’s ANOVA and Wilcoxon signed ranks tests were used for post-hoc comparisons of number of yawns per minute across the baseline, yawn, gape and nose-wipe phases. Cochran’s Q and McNemar tests were used for binomial analyses. Moreover, Mann–Whitney *U* tests were used to compare rate of yawning by age and sex and Kruskal-Wallis tests to test for order effects. Data were analysed using SPSS Statistics 21 for Macintosh (SPSS, IBM Inc.). Values reported are mean ± SEM, and all tests were two-tailed and significance levels set at α = 0.05.

### Ethics Statement

Experimental procedures were non-invasive and complied with the ethical guidelines of the Pan African Sanctuary Alliance and Animal Behavior Society Guidelines for the Use of Animals in Research. The ethics of the experimental setup was evaluated by the Regional Ethics Committee at Uppsala District Court in Sweden, and the board of Tacugama Chimpanzee Sanctuary (in Sierra Leone) reviewed and approved the study.

## Results

### Yawning

**Figure 1:**
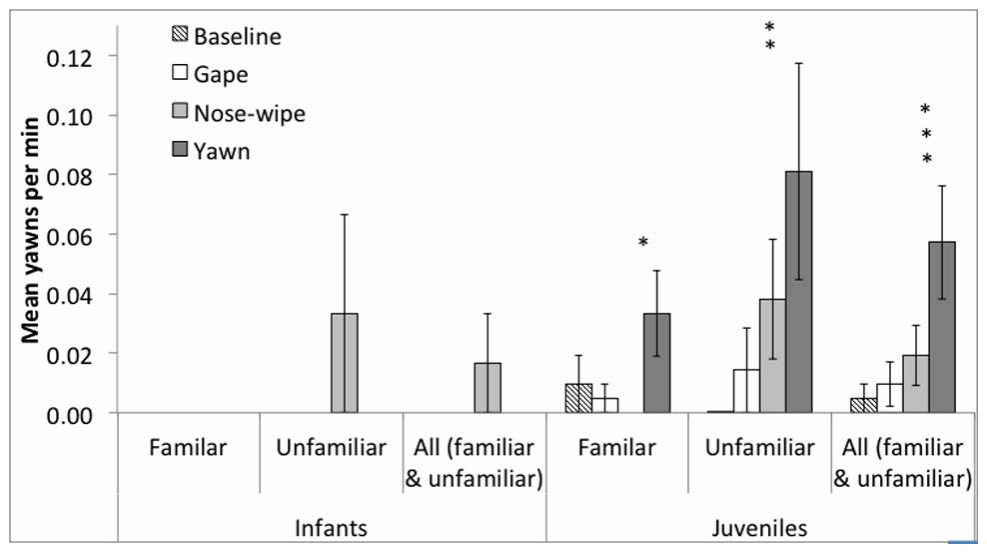
Rate of yawning across conditions. Yawns per minute (± SEM) across the baseline, gape, nose-wipe and yawn conditions for trials with infants and juveniles with a familiar and unfamiliar model, as well as data from the familiar and unfamiliar conditions collapsed.

The yawn condition elicited 24 yawns from the juvenile chimpanzees and none from the infants, a statistically significant difference (Mann-Whitney U test: *U* = 66, *z* = 2.77, *P* = 0.006). Analysis of the number of yawns per minute revealed a significant effect of age (GLMM: *F* = 7.80, df = 1, *P = *0.008), and an interaction between age and model behaviour (none/baseline, yawn, nose-wipe, gape: *F* = 4.54, df = 3, *P = *0.011, Figure 1). Model behaviour only approached significance as an independent predictor (*F* = 2.70, df = 3, *P = *0.057), while model familiarity was non-significant (*F* = 2.77, df = 1, *P = *0.109). A difference in the number of juvenile yawns per minute across the baseline (0.00), yawn (0.06), nose-wipe (0.02) and gape phases (0.01, Friedman test: *X* ^2^_(3)_ = 16.64, *P* = 0.001), reflected that juveniles were more likely to yawn in the yawn phase than all other phases (Wilcoxon test: yawn vs. baseline: N = 33, *z* = 2.34, *P* = 0.019; yawn vs. gape: *N* = 33, *z* = 2.68, *P* = 0.007; yawn vs. nose-wipe: *N* = 33, *z* = 2.55, *P* = 0.011). Both the familiar (Friedman test: *X* ^2^_(3)_ = 8.43, *P* = 0.038) and unfamiliar model (*X* ^2^_(3)_ = 13.02, *P* = 0.004) elicited significant differences in juvenile yawning across the conditions, though with slight differences in the patterns of results (see figure 1). Juveniles produced more than twice as many yawns in response to unfamiliar (mean = 0.81±0.36) compared to familiar yawn stimuli (mean = 0.33±0.14), although the difference was not statistically significant (N = 21, *z* = 0.88, *P* = 0.379). Overall, across conditions, juvenile chimpanzees yawned at a higher frequency than infants (infants: mean = 0.33±0.33; juveniles: mean = 1.76±0.63, Mann–Whitney test: *U* = 62.00, *z* = 2.68, *P* = 0.007). Moreover, juveniles produced twice as many yawns in the yawn phase (n = 16, mean = 0.76±0.24) compared to the post-yawn phase (n = 8, mean 0.38±0.20), although the difference did not reach statistical significance (Wilcoxon test: n = 21, *z* = 1.64, *P* = 0.131).

Analysis of the number of chimpanzees the yawned (rather than the number of yawns per minute across the conditions) also revealed a significant difference in the number of juvenile chimpanzees that yawned across the yawn (48%), nose-wipe (19%) and gape phases (10%, Cochran’s Q; Q_(2)_ = 9.46, *P* = 0.009). Pairwise comparisons showed that more juvenile chimpanzees yawned in the yawn than gape phase (Q_(1)_ = 2.95, *P* = 0.009), while the difference between the yawn and nose-wipe phase only approached significance (Q_(1)_ = 2.22, *P* = 0.080). An equivalent number of juveniles yawned in the gape and nose-wipe conditions (Q_(1)_ = 0.74, *P* = 1). The behaviour (yawn, nose-wipe, gape) of both the familiar and unfamiliar models elicited differences in the number of juveniles that yawned (Cochran’s Q: Familiar: Q_(2)_ = 7.00, *P* = 0.03; Unfamiliar: Q_(2)_ = 6.33, *P* = 0.042), though with slight differences. Pairwise comparison showed that the familiar model evoked yawns from more juveniles when she yawned than nose-wiped (Q_(1)_ = 2.50, *P* = 0.037), while the unfamiliar model evoked more yawns when she yawned than gaped (Q_(1)_ = 2.50, *P* = 0.037). In contrast, there was no difference in the number of juveniles that yawned in the yawn and gape phase when the behaviours were performed by a familiar model (Q_(1)_ = 2.00, *P* = 0.137), and in the yawn and nose-wipe phases when the behaviours were performed by an unfamiliar model (Q_(1)_ = 1.00, *P* = 0.952). Moreover, both the familiar and unfamiliar models evoked yawns in a similar number of juveniles in the gape and nose-wipe phases (familiar: Q_(1)_ = 0.50, *P* = 1; Unfamiliar: Q_(1)_ = 1.50, *P* = 0.401). An equal number of juveniles yawned to familiar and unfamiliar yawn stimuli (McNemar test: *P = *1). Finally, yawn frequency did not differ between the sexes (Mann–Whitney test: *U* = 108, *z* = 1.20, *P* = 0.343) and there were no order effects (i.e., the frequency of yawning in the yawn condition was not influenced by order of presentation, with yawn condition presented as first, second or third condition after the baseline: familiar model: Kruskal-Wallis test: *H* = 0.37, *P* = 0.896; unfamiliar model: *H* = 5.17, *P* = 0.075).

### Nose-wiping

**Figure 2:**
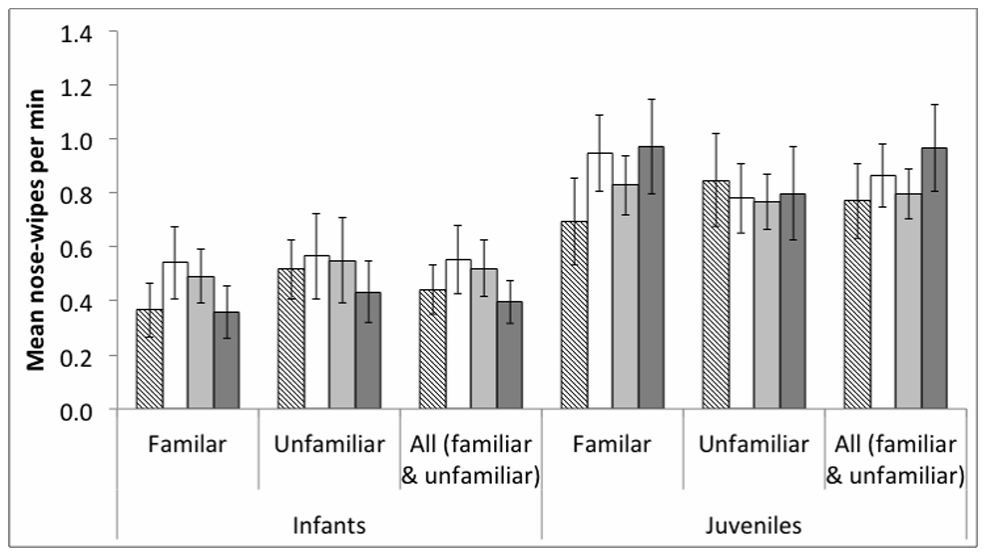
Rate of nose-wiping across conditions. Nose-wipes per minute (± SEM) across the baseline, gape, nose-wipe and yawn conditions for trials with infants and juveniles with a familiar and unfamiliar model, as well as data from the familiar and unfamiliar conditions collapsed.

Analysis of the number of chimpanzee nose-wipes per minute revealed no effect of either model behaviour (none/baseline, yawn, nose-wipe, gape: GLMM: *F* = 0.57, df = 3, *P = *0.648), model familiarity (*F* = 0.21, df = 1, *P = *0.651), or age (*F* = 6.74, df = 1, *P = *0.067), although age approached significance (figure 2). Further analysis of the influence of age, showed, that (as for yawning, see above), juvenile chimpanzees nose-wiped at a higher frequency than infants throughout the experiment (infants: mean = 33.83±5.85, juveniles: mean = 60.29±8.17, Mann-Whitney test: *U* = 67.50, *z* = 2.19, *P* = 0.028). Moreover, juveniles produced more nose-wipes in the nose-wipe (mean = 9.43±1.16) than post-nose-wipe phase (mean = 6.52±0.96, n = 21, *z* = 2.54, *P* = 0.009), while there was no similar effect for infants (nose-wipe phase: mean = 5.92±1.27, post-nose-wipe phase: mean = 4.50±1.28, Wilcoxon test: *z* = 1.07, *P* = 0.315). With respect to the number of chimpanzees the nose-wiped across conditions (rather than the number of nose-wipes per minute), all but one chimpanzee (an infant), nose-wiped in response to the models’ yawns, nose-wipes and gapes, respectively. Moreover, equal numbers of chimpanzees nose-wiped when the familiar and unfamiliar models, respectively, yawned, nose-wiped and gaped (Cochran’s Q: familiar: Q_(2)_ = 2.00, *P* = 0.368; unfamiliar: Q_(2)_ = 2.00, *P* = 0.368).

## Discussion

The current study is the first to demonstrate the existence of cross-species contagious yawning in chimpanzees. It further shows that, like humans [3], [42] and dogs [12], chimpanzees are subject to a developmental increase in susceptibility to yawn contagion. Viewing a human yawn elicited yawning in 48 per cent of juvenile chimpanzees, while infants were immune to contagion (no infant yawned in response to human yawns). This age effect is consistent with the suggestive results of a previous study failing to elicit CY in three infant chimpanzees accompanying their mothers, when these viewed videoed conspecific yawns [6]. Regardless of the medium (live or videoed), infant chimpanzees seem immune to yawn contagion. The results, moreover, suggest that heterospecific yawn contagion in dogs [11], [12] is not necessarily a function of the social domestication of this species, but that contagion in chimpanzees and dogs may reflect either prolonged ontogenetic interaction with humans or general attention to the emotional and/or physical states of others, regardless of the species, to which they belong.

There is increasing evidence that mirror neuron networks may be recruited for yawn contagion, although the extent thereof remains debated [5], [45], [75]–[79]. While however, children [80], chimpanzees [81] and macaques, *Macaca mulatta* [82] exhibit neonatal imitation (thought to be underpinned by mirror neurons [83]), young chimpanzees and children are not subject to yawn contagion (children reach adult-like levels only by around 12 years of age [3]). Whether CY may be part of the group of ‘contagious’ mouth behaviours that occur during the short window of neonatal imitation (and subsequently disappears) nonetheless remains untested. If CY does not appear during the restricted time span of neonatal imitation, this suggests that CY involves, at least in part, other mechanisms than mirror neurons, or that the action understanding, that mirror neurons underpin, may improve with experience (see [84]), and thus age.

The distribution of CY across and within species has been suggested to be consistent with the claim, that yawn contagion shares its mechanism with the capacity for theory-of-mind [4], [43]. Nonetheless, yawn contagion in young dogs (from 7 mts of age) has been interpreted as evidence suggesting that CY is underlain by processes less complex than *cognitive empathy* [12], which is considered to involve theory-of-mind-like attribution and perspective-taking. Indeed, there is little empirical evidence of theory-of-mind attribution in young dogs or juvenile chimpanzees. Interestingly, there is some evidence of perspective-taking in young dogs at 8 mts of age [85], and in chimpanzees the emergence of CY coincides with that of perspective-taking (around 4.5 years, as examined by performance on the ‘guesser-knower’ paradigm [86]).

Overall, the existence of CY in non-human species, and the development of CY in humans and other species, is consistent with the notion that the development of *affective empathy*, and an emerging capacity for perspective-taking (as well as increased attention to and improvement in the identification of others’ affective states) is sufficient to explain the distribution of yawn contagion, ontogenetically and phylogenetically. The comparatively late emergence of CY relative to evidence of *affective* empathy in children below 4 years of age, suggests that CY may rely on the development of other and interacting capacities (such as perspective-taking, attention to and identification of others emotional states). While CY co-emerges temporally with *cognitive empathy* in children [3], [42]) and correlates with adult performance on theory-of-mind-tasks in humans [4], CY is not a reliable marker of the presence of *cognitive empathy* in a species. A lower-level hypothesis suggests that susceptibility to yawn contagion relies on attentional states and biases [27]. Experimental studies demonstrating a developmental effect on yawn contagion in non-human animals (the present experiment and [12]) have, however, controlled for the attentional focus of both younger and older individuals.

On a proximate level, the immunity of infant chimpanzees to CY may indicate a developmental immaturity of socio-cognitive skills and/or neural networks involved in processing social information [10]. That is, it may reflect developmental changes in action-understanding (based on mirror neurons, which appear to acquire their properties through experience [86]), perspective-taking and/or attention to and identification of others’ affective states. On an ultimate level, there may be a less strong selective pressure for immature individuals to synchronise and coordinate behaviour with others, given their lesser roles in group-decision processes.

In contrast to yawning, there was no contagion effect for nose-wiping. Juvenile chimpanzees were, however, more likely to wipe their nose during the 5-min interval, where they viewed a model nose-wipe, than in the subsequent 5 minutes (a similar, non-significant, trend was found for yawning). There was however, a (non-significant) trend for juvenile chimpanzees to nose-wipe at a higher frequency across all conditions than infants. Thus, while nose-wiping might be (low-level) contagious in humans (similar to itching [70], [71]), it is not so for chimpanzees, when performed by a human model. Moreover, in line with recent research suggesting that human itching is not related to individual levels of empathy [71], we found no evidence that chimpanzee nose-wiping was related to empathy with the observed model (operationalized as familiarity).

Contrary to prediction, but consistent with findings for human children [46], young dogs [12] and adult chimpanzees [8], we found no evidence that emotional closeness with the model increased the susceptibility of young chimpanzees to CY. While ‘liking’ a model increases spontaneous, non-conscious facial mimicry in adult humans [64] and has been suggested to facilitate imitation [65], the present study does not provide evidence in support of this. In contrast, Chartrand and Bargh [47] have shown that, in humans, social motivations, such as the desire to affiliate or bond with another, modulate non-conscious mimicry. The (albeit statistically non-significant) tendency of juvenile chimpanzees to yawn more in response to the yawns by the *unfamiliar* (than familiar) model may suggest that the ‘chameleon effect’ [47] operates in heterospecific yawn contagion contexts. Young chimpanzees may be motivated to smooth interactions with unfamiliar human partners. Overall, the results open for at least three testable hypotheses: (1) A potential social modulatory effect on CY emerges only at later stages of development [12]. While juvenile chimpanzees (present results) and young dogs [12] exhibit CY, neither have shown a social modulatory effect on CY, whereby familiarity with a human model has influenced susceptibility to contagion. In contrast, there is some evidence that CY in adult members of these species is influenced by familiarity [7], [54], [13]. Further examination of this hypothesis might test the effect of model familiarity on CY in young children, that otherwise do exhibit CY (i.e. children aged 4–6 yrs.). (2) Emotional closeness with a model does not affect CY in chimpanzees. While one study has shown that conspecific CY in adult chimpanzees is influence by *model familiarity* (in- and out-group membership [7]), another has failed to evince an effect of *relationship quality* with familiar conspecifics [8] (used as a proxy for emotional closeness). A further test of the effect of relationship quality on chimpanzee CY might use observations of yawns in response to spontaneous conspecific yawns (a methodology that has shown a familiarity effect in adult humans [26], bonobos [9] and baboons [10]). (3) Heterospecific yawns do not elicit a familiarity effect on CY in chimpanzees. Adult chimpanzees have only been tested on yawn contagion when viewing the yawns of *conspecifics*, while young chimpanzees have only been explicitly tested with respect to *heterospecific* yawn contagion. Given that chimpanzees typically engage in competitive, or even hostile, relationships with unfamiliar conspecifics, but do not automatically do so with unfamiliar humans, the familiarity effect on yawn contagion in adult chimpanzees, may only apply when chimpanzees view the yawns of conspecifics. That is, adult chimpanzees may apply ‘targeted empathy’ to interactions with conspecifics, while they apply a more generalised form of empathy to interactions with humans, who they rarely engage in competition with and mostly experience as cooperative (in e.g. food provision contexts). Further research is thus required to ascertain the mechanisms underlying the variable results in studies of CY across ages and species.

## Supporting Information

Table S1 Participant details (name, age class, estimated age – determined by the sanctuary veterinarians based on weight and dental data –, housing, time spent at the sanctuary and sex).
(DOCX)

Table S2 Order of presentation of the conditions (model familiarity and model behaviour).
(DOCX)

Video S1 **Baseline, yawning, nose-wiping and gaping conditions with the familiar and unfamiliar models (both experimenters have provided written informed consent, as outlined in the PLOS consent form, to publication of their image).**
(M4V)
