# Supplementary material for: Chimpanzees Show a Developmental Increase in Susceptibility to Contagious Yawning: A Test of the Effect of Ontogeny and Emotional Closeness on Yawn Contagion
Source: PLoS One. 2013 Oct 16;8(10):e76266. doi: 10.1371/journal.pone.0076266 (PMC3797813; doi:10.1371/journal.pone.0076266)
Supplement: Table S1 — Participant details (name, age class, estimated age – determined by the sanctuary veterinarians based on weight and dental data –, housing, time spent at the sanctuary and sex). (DOCX) [file pone.0076266.s001.docx]

| **SS** | **Name** | **Age category** | **Age (yr.)** | **D.O.B. (estimated)** | **Housing (group size)** | **Time at sanctuary** | **Sex** |
| --- | --- | --- | --- | --- | --- | --- | --- |
| 1 | Sara | Infant | 1.1 | Oct. 2009 | Solitary | 5 | F |
| 2 | A.J. | Infant | 1.5 | Apr. 2009 | Solitary | 1 | M |
| 3 | Tompey | Infant | 1.5 | June 2009 | Solitary | 9 | F |
| 4 | Kangari | Infant | 2.5 | June 2008 | Solitary | 12 | M |
| 5 | Bainyaa | Infant | 3 | Aug. 2007 | 14 | 25 | F |
| 6 | Gaura | Infant | 3 | Jan. 2007 | 14 | 34 | M |
| 7 | Mac | Infant | 3 | June 2007 | 14 | 28 | M |
| 8 | Tombo | Infant | 3 | July 2007 | 14 | 28 | F |
| 9 | Delilah | Infant | 3 | Nov. 2007 | 2 | 12 | F |
| 10 | Samson | Infant | 3 | Dec. 2007 | 2 | 20 | M |
| 11 | Bimbo | Infant | 4 | Jan. 2006 | 14 | 28 | M |
| 12 | Benita | Infant | 4 | Dec. 2006 | Solitary | 10 | F |
| 13 | Chica | Juvenile | 5 | Feb. 2005 | 14 | 57 | F |
| 14 | Cim | Juvenile | 5 | Apr. 2005 | 22 | 55 | M |
| 15 | Jerusalem | Juvenile | 5 | Jan. 2005 | 22 | 46 | F |
| 16 | Alex | Juvenile | 6 | Apr. 2004 | 14 | 43 | M |
| 17 | Bidi | Juvenile | 6 | Mar. 2004 | 14 | 36 | F |
| 18 | Spana | Juvenile | 6 | Jun. 2004 | 14 | 50 | M |
| 19 | Nita | Juvenile | 6 | Feb. 2004 | 14 | 52 | F |
| 20 | Simon | Juvenile | 6 | Jun. 2004 | 22 | 62 | M |
| 21 | Natasha | Juvenile | 6 | Oct. 2004 | 22 | 61 | F |
| 22 | Zeelie | Juvenile | 6 | Jan. 2004 | 22 | 62 | M |
| 23 | Bebi | Juvenile | 7 | Jan. 2003 | 22 | 66 | F |
| 24 | Grant | Juvenile | 7 | Apr. 2003 | 22 | 60 | M |
| 25 | Jimmy | Juvenile | 7 | Jun. 2003 | 22 | 66 | M |
| 26 | Junior | Juvenile | 7 | Jun. 2003 | 22 | 76 | M |
| 27 | Kouze | Juvenile | 7 | Aug. 2003 | 22 | 64 | M |
| 28 | Mary | Juvenile | 7 | Aug. 2003 | 22 | 38 | F |
| 29 | Nyawa | Juvenile | 7 | Dec. 2003 | 22 | 71 | F |
| 30 | Peke | Juvenile | 7 | Jun. 2003 | 22 | 57 | M |
| 31 | Umno | Juvenile | 7 | Jan. 2003 | 22 | 67 | M |
| 32 | Jane | Juvenile | 8 | Jan. 2002 | 22 | 52 | F |
| 33 | Joko | Juvenile | 8 | Jan. 2002 | 22 | 52 | M |
