# Supplementary material for: Chimpanzees Show a Developmental Increase in Susceptibility to Contagious Yawning: A Test of the Effect of Ontogeny and Emotional Closeness on Yawn Contagion
Source: PLoS One. 2013 Oct 16;8(10):e76266. doi: 10.1371/journal.pone.0076266 (PMC3797813; doi:10.1371/journal.pone.0076266)
Supplement: Table S2 — Order of presentation of the conditions (model familiarity and model behaviour). (DOCX) [file pone.0076266.s002.docx]

| **Participant ID** | **Participant** | **Ver-sion** | **Order: Familiarity^1^** | **Order: Behaviour** | | |
| --- | --- | --- | --- | --- | --- | --- |
|  |  |  |  | **1** | **2** | **3** |
| 3 | Tompey | 1 | 1 | Yawning | Nose-wiping | Gaping |
| 10 | Samson | 1 | 1 | Yawning | Nose-wiping | Gaping |
| 31 | Umno | 1 | 1 | Yawning | Nose-wiping | Gaping |
| 28 | Mary | 1 | 1 | Yawning | Nose-wiping | Gaping |
| 15 | Jerusalem | 1 | 1 | Yawning | Nose-wiping | Gaping |
| 25 | Jimmy | 1 | 1 | Yawning | Nose-wiping | Gaping |
| 9 | Delilah | 2 | 1 | Nose-wiping | Gaping | Yawning |
| 12 | Benita | 2 | 1 | Nose-wiping | Gaping | Yawning |
| 30 | Peke | 2 | 1 | Nose-wiping | Gaping | Yawning |
| 17 | Bidi | 2 | 1 | Nose-wiping | Gaping | Yawning |
| 20 | Simon | 2 | 1 | Nose-wiping | Gaping | Yawning |
| 1 | Sara | 3 | 1 | Gaping | Yawning | Nose-wiping |
| 11 | Bimbo | 3 | 1 | Gaping | Yawning | Nose-wiping |
| 6 | Gaua | 3 | 1 | Gaping | Yawning | Nose-wiping |
| 23 | Bebi | 3 | 1 | Gaping | Yawning | Nose-wiping |
| 21 | Natasha | 3 | 1 | Gaping | Yawning | Nose-wiping |
| 4 | Kangari | 4 | 2 | Yawning | Nose-wiping | Gaping |
| 5 | Bainya | 4 | 2 | Yawning | Nose-wiping | Gaping |
| 19 | Nita | 4 | 2 | Yawning | Nose-wiping | Gaping |
| 14 | Cim | 4 | 2 | Yawning | Nose-wiping | Gaping |
| 29 | Nyawa | 4 | 2 | Yawning | Nose-wiping | Gaping |
| 13 | Chica | 4 | 2 | Yawning | Nose-wiping | Gaping |
| 2 | AJ | 5 | 2 | Nose-wiping | Gaping | Yawning |
| 8 | Tombo | 5 | 2 | Nose-wiping | Gaping | Yawning |
| 24 | Grant | 5 | 2 | Nose-wiping | Gaping | Yawning |
| 27 | Kouze | 5 | 2 | Nose-wiping | Gaping | Yawning |
| 22 | Zeelie | 5 | 2 | Nose-wiping | Gaping | Yawning |
| 32 | Jane | 5 | 2 | Nose-wiping | Gaping | Yawning |
| 18 | Spana | 6 | 2 | Gaping | Yawning | Nose-wiping |
| 7 | Mac | 6 | 2 | Gaping | Yawning | Nose-wiping |
| 16 | Alex | 6 | 2 | Gaping | Yawning | Nose-wiping |
| 26 | Junior | 6 | 2 | Gaping | Yawning | Nose-wiping |
| 33 | Joko | 6 | 2 | Gaping | Yawning | Nose-wiping |

^1^ 1 = Familiar model first; 2 = Unfamiliar model first
